# Determining IFI44 as a key lupus nephritis’s biomarker through bioinformatics and immunohistochemistry

**DOI:** 10.1080/0886022X.2025.2479575

**Published:** 2025-03-18

**Authors:** Yue Tan, Xueyao Wang, Deyou Zhang, Jiahui Wang, Shuxian Wang, Jinyu Yu, Hao Wu

**Affiliations:** a Department of Nephrology, The First Hospital of Jilin University, Changchun, China; b Department of Critical Care Medicine, The First Hospital of Jilin University, Changchun, China; c Department of Renal Pathology, The First Hospital of Jilin University, Changchun, China

**Keywords:** Lupus nephritis, interferon-induced protein 44, gene set enrichment analysis (GSEA), clinicopathological significance

## Abstract

**Background:**

Lupus nephritis (LN) emerges as a severe complication of systemic lupus erythematosus (SLE), significantly affecting patient survival. Despite improvements in treatment reducing LN’s morbidity and mortality, existing therapies remain suboptimal, emphasizing the necessity for early detection to improve patient outcomes.

**Methods:**

This study employs bioinformatics and machine learning to identify and validate potential LN biomarkers using immunohistochemistry (IHC). It explores the relationship between these biomarkers and the clinical and pathological characteristics of LN, assessing their prognostic significance. The research provides deeper mechanistic insights by employing Gene Set Enrichment Analysis (GSEA), Gene Ontology (GO), and Kyoto Encyclopedia of Genes and Genomes (KEGG) analyses. Additionally, the study characterizes the immune profiles of LN patients through the CIBERSORT algorithm, focusing on the role of interferon-inducible protein 44 (IFI44) as a key biomarker.

**Results:**

IFI44 shows elevated expression in LN-affected kidneys, compared to healthy controls. The levels of IFI44 positively correlate with serum creatinine and the Systemic Lupus Erythematosus Disease Activity Index (SLEDAI) and inversely with serum complement C3 and initial estimated glomerular filtration rate (eGFR).

**Conclusion:**

IFI44 is identified as a promising biomarker for LN, offering potential to refine the assessment of disease progression and predict clinical outcomes. This facilitates the development of more personalized treatment strategies for LN patients.

## Introduction

As a complex autoimmune condition, systemic lupus erythematosus (SLE) is shaped by a blend of environmental, immunological, and genetic factors, manifesting across multiple bodily systems [1]. Among the serious complications of SLE, lupus nephritis (LN) is notably common. Lupus nephritis (LN) significantly heightens the incidence of adverse health outcomes among patients with systemic lupus erythematosus (SLE), with approximately 20% advancing to end-stage renal disease [2]. Enhancing treatment outcomes and lowering mortality rates necessitate improved monitoring, evaluation of disease activity, and prognosis assessment. Biomarkers are pivotal in accurately gauging disease activity, aiding physicians in tailoring personalized treatment plans [3]. Despite advancements, the reliability of indicators for routine monitoring of lupus nephritis (LN) remains uncertain. At present, invasive renal biopsy stands as the primary method for diagnosing and gauging the severity of the condition [4]. Two National Institutes of Health (NIH) indices, namely Chronicity Index (CI) and Activity Index (AI) have been used for assessing renal prognosis in LN [5,6]. However, their capability to foresee long-term prognosis from the initial renal biopsy stage needs more research [7]. Thus, there is a pressing need to explore more precise biomarkers for early LN detection and patient monitoring. Recent advancements in bioinformatics have enabled the discovery of novel diagnostic biomarkers through differential gene expression analysis [8,9]. In our study, we employed bioinformatics techniques to identify interferon-inducible protein 44 (IFI44) as a biomarker for LN. A comprehensive biosignature analysis by Lingling Shen et al. highlighted five central genes—DDX60, RSAD2, HERC5, IFIT3, and IFI44—as significant in LN pathogenesis. Among these, IFI44 demonstrated notable diagnostic efficacy, confirmed through qPCR and ELISA validation [10]. Consistent with our study, IFI44 is recognized as a noteworthy biomarker deserving further exploration. Recent studies have linked anti-IFI44 antibodies to various systemic autoimmune diseases, such as Sjögren’s syndrome [11], rheumatoid arthritis [12], dengue fever [13], psoriasis [14], and SLE [15]. Despite the strong link between IFI44 expression and LN, there is a lack of renal biopsy data on IFI44 expression in patients with lupus nephritis (LN).

Recognizing IFI44’s potential significance in assessing the progression of lupus nephritis (LN), we launched a study to explore its value in evaluating disease outcomes. Our research involved gathering data from patients diagnosed with LN, confirming IFI44 levels in kidney tissue through immunohistochemical staining, and investigating its association with LN progression and prognosis. Moreover, GSEA method also called ‘gene set enrichment analysis’, was used to explore potential mechanisms involving IFI44 in LN, alongside assessing the relationship between IFI44 expression and immune cell infiltration.

## Materials and methods

Using the GSE32591 dataset, we obtained microarray data including samples from 29 healthy kidney tissues (Normal) and 64 kidney tissues affected by lupus nephritis (LN). The transcriptional levels in LN patients were quantified using GPL14663 data.

Furthermore, the limma package was employed to identify genes showing differential expression (DE) between the healthy individuals and those with lupus nephritis (LN), using criteria of a P value < 0.05 and an absolute log fold change (|logFC|) > 0.585. Using R language, volcano plots were generated with ggplot2 package, while heatmaps were created with pheatmap package. The heatmaps displayed the logFCs, highlighting the 25 most significantly upregulated and 25 most significantly downregulated genes. To further investigate gene expression differences between the normal individuals and LN patients, a boxplot for the IFI44 gene was created using the ggboxplot function in R with distinct colors representing the lupus nephritis and normal groups. Additionally, the statistical difference between groups was determined with the stat_compare_means function, indicating significance levels with symbolic markers. Using the pROC package, the diagnostic accuracy was assessed by calculating the performance metric known as the area under the curve (AUC) of the subject’s work-specific ROC curve.

### Patient and sample collection

All tissue samples for immunohistochemistry (IHC) staining were collected from 48 patients with lupus nephritis (LN) and 10 control individuals between May 2016 and August 2020 at the First Hospital of Jilin University. We received the written informed consent from participants in this study. The clinical diagnosis of patients with lupus nephritis (LN) was confirmed through renal biopsy, also with a specific index called SLEDAI. Inclusion Criteria: (1) Patients must be 18 years or older at the time of lupus nephritis (LN) diagnosis. (2) Renal biopsy specimens must contain 10 or more glomeruli. (3) Patients must have relatively complete follow-up data available. Exclusion Criteria: (1) Presence of other glomerular diseases. (2) Presence of coexisting renal tumors. All patients included in the study met the 1997 American College of Rheumatology revised classification criteria for systemic lupus erythematosus (SLE) and were confirmed to have LN through pathological examination using light microscopy, immunofluorescence, and transmission electron microscopy.

Several tissue samples obtained from six patients diagnosed with minimal change disease (MCD) were used as controls for renal disease. Normal control (NC) tissues were sourced from four discarded kidney tissues that were confirmed healthy through comprehensive evaluations involving immunofluorescence staining and microscopic examination under routine light microscope, and even with electron microscopy. Our research complied with the ethical principles outlined in the Declaration of Helsinki, with an approvement of the Ethics Committee in our hospital.

### Clinical parameters

We extracted detailed clinical and laboratory examination information from the electronic medical records of lupus nephritis (LN) patients stored in our hospital. This information included age, gender, hypertension status, the SLEDAI, occurrences of hematuria and leukocyturia, serum levels of creatinine, C3, C4, and albumin, as well as 24-h proteinuria and glomerular filtration rate.

### Renal immunohistochemical (IHC) staining

Renal sections obtained from normal controls, disease controls, and LN patients were subjected to a series of preparatory procedures. First, the deparaffinization of tissue sections was performed using chemical including xylene and ethanol, then using different concentration of ethanol for their rehydration. Antigen retrieval was then performed by heating (5 min) in either Tris/EDTA buffer (1x, pH 8.5) or citrate buffer (0.01 M, pH 6.0), and endogenous peroxidase activity was blocked using 3% H2O2 for a brief period. Immune blotting was performed through overnight incubation (4 °C) with primary antibody (anti-IFI44, ab169788, Abcam, Cambridge, UK). One hour-incubation with 2^nd^ antibody (goat anti-mouse/rabbit, Maixin Company, Fuzhou, China) was performed on next day at room temperature. The results were visualized using the chromogenic substrate DAB (3,3-diaminobenzidine), which is included in a detection kit from Maixin Company, (Fuzhou, China). Contrast staining was also applied using hematoxylin. Finally, the results were examined with the OLYMPUS cellSens Entry microscopy system. A specific software (Image-Pro Plus, v6.0 from Media Cybernetics, Dallas, TX, USA) was used for analyzing IHC data, including mean optical density (integrated optical density/area). In a blinded manner, quantitative analysis involved evaluating all glomeruli within each section and tubulointerstitium in every section in high-power fields.

### Multiplex immunofluorescence (IF) staining

Kidney tissue samples underwent antigen retrieval by a method as mentioned above. The slides were then incubated with 4 types of primary antibodies from Maixin Company (Fuzhou, China), including anti-CD20, anti-CD3, anti-CD31 and anti-CD68. Other primary antibodies, such as anti-myeloperoxidase (MPO), anti-integrin-8, anti-Wilms Tumor protein (WT1), and anti-IFI44 were purchased from Bio-techne (Minnesota, USA), Sigma-Aldrich (St. Louis, USA), and Abcam (Cambridge, UK), separately. All of those antibodies were used separately or in sequence.

The immunofluorescence signals were enhanced using a multiplex system developed with the TSA-RM 20 U kit (Panovue, Beijing, China). The enhancement involved a 30-min incubation at room temperature using goat anti-mouse/rabbit-HRP. The samples were incubated with a fluorophore solution at a 1:100 dilution, with a DAPI (4, 6-diamidino-2-phenylindole) counterstaining of nuclei. Fluorescence images were then captured using a confocal microscope (Olympus FV-3000, Japan).

### GO, KEGG and GSEA analyses for IFI44

We investigated how IFI44 expression relates to function in LN patients by categorizing individuals into groups with high and low IFI44 expression levels according to their median value. We also applied the GSEA method to analyze the differences in IFI44 expression levels between these groups. Meanwhile, we performed gene annotation and functional classification using Gene Ontology (GO) framework, in which there are three categories: BP (biological processes), CC (cellular components), and MF (molecular functions). Those categories underwent functional enrichment analysis. The metabolic pathways associated with these genes were investigated by KEGG analysis. This analysis shed light on the metabolic pathways and biological processes the genes are involved in, thereby enriching our understanding of the intricate biological systems. Furthermore, R package ‘clusterProfiler’ was used for the gene enrichment analysis to pinpoint their biological functions and associated pathways.

### Infiltration of immune cells

We employed bioinformatics tools, such as the CIBERSORT algorithm, to analyze immune characteristics differences between LN patients and healthy individuals. Moreover, we visualized the patterns of infiltration of 22 different types of immune cells between LN patients and healthy individuals using violin plots generated with the R package ‘ggplot2’. Additionally, the association of IFI44 expression with the infiltration patterns of immune cells was examined using Spearman’s rank correlation, a statistical method. We then used the R package ‘ggplot2’ to visualize the analysis results.

### Statistical analysis

The software used for data analysis in this study are GraphPad Prism 8 (San Diego, USA) and SPSS version 20 (Chicago, USA). We assessed the statistical differences in continuous measurement data between groups using methods such as Student’s t-test or ANOVA. Pearson’s test was used for analyzing the correlations between parametric data, while Spearman’s test was applied for nonparametric variables.

## Results

### IFI44 is a promising indicator for LN diagnosis

**Figure 1.**
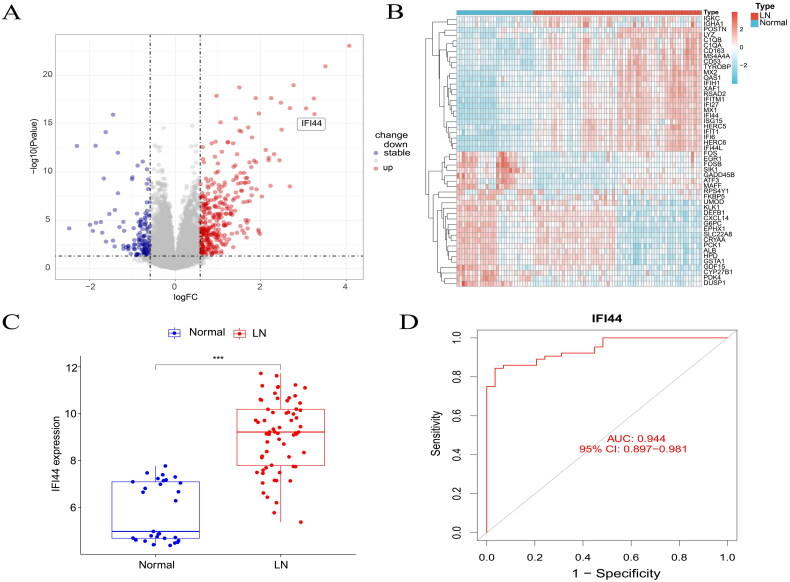
Identification of critical indicators of lupus nephritis (LN). (A) Visualization of DEGs in LN by a volcano plot. This plot illustrates upregulated genes in red and downregulated genes in green, providing a clear visual differentiation. (B) Heat maps were generated to depict the top 25 upregulated and 25 downregulated genes, offering a detailed representation of gene expression patterns. (C) The diagnostic power of IFI44 levels was assessed based on its performance in the training set (ROC curve). (D) A thorough analysis was undertaken to explore the fluctuations in IFI44 expression within the training dataset.

Within GSE32591 dataset, 64 samples from LN patients and 29 from normal individuals were analyzed, revealing 487 differentially expressed genes (DEGs) in all. Of these, 341 were significantly upregulated, and 146 were downregulated. The volcano plot highlights the significant upregulation of the IFI44 gene in LN patient samples (Figure 1A). Additionally, a heatmap was generated by sorting the logFC and selecting 50 genes with the most significant up- and down-regulation. This heatmap further confirms the notable upregulation of the IFI44 gene in LN patient samples (Figure 1B).

Moreover, the expression levels of the IFI44 gene were plotted across different subgroups of LN patients and the normal individuals (Figure 1C). The ROC curve was also plotted to assess diagnostic performance (Figure 1D). IFI44 expression was notably heightened among patients diagnosed with LN. The IFI44 gene demonstrated significant diagnostic value in distinguishing LN patients from normal individuals, exhibiting its high performance (ROC AUC = 0.944, 95% CI: 0.897–0.981).

### IFI44 is significantly overexpressed in the renal tissue of patients with LN

**Figure 2.**
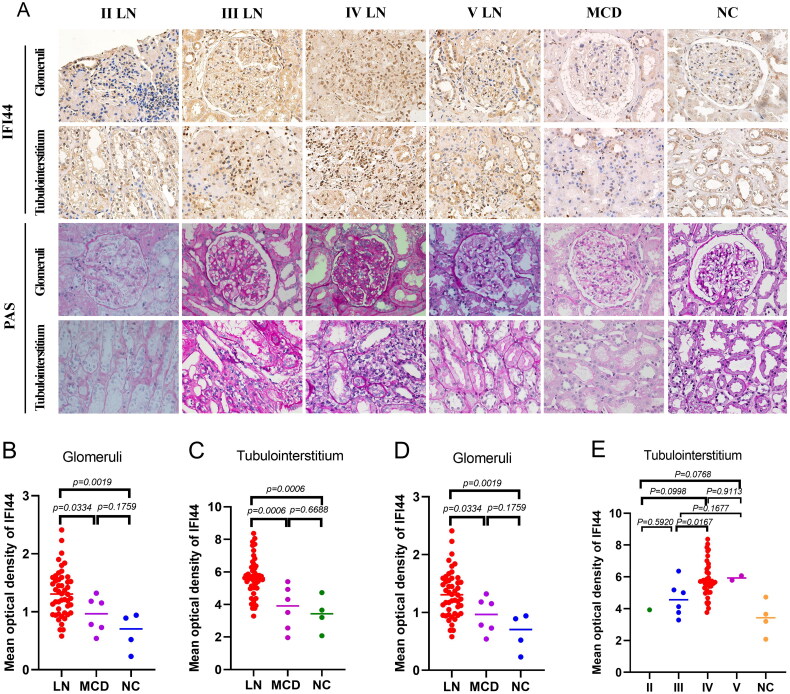
Immunohistochemical results of IFI44 expression in renal tissues. (A) This panel displays immunohistochemical staining of IFI44 in both the tubulointerstitium (below) and glomeruli (above) at 400x magnification across different groups: those with lupus nephritis (LN), minimal change disease (MCD), and normal controls (NC). (B) This graph shows the average optical density measurements of IFI44 specifically in the glomeruli, highlighting differences between the studied groups. (C) Similarly, this graph presents AOD of tubulointerstitial IFI44 expression. Next two panels display IFI44 expression AOD in both glomerular (D) and tubulointerstitial (E) regions among different classifications of LN pathology, providing insights into IFI44 expression levels within different severities of the disease. Note: AOD, average optical density

Immunohistochemistry (IHC) staining on renal tissue biopsy samples was conducted in our study. The samples were collected from 48 patients diagnosed with LN, 6 subjects with MCD (minimal change disease) and 4 NC samples (normal control). Supplementary Table 1 provides the pathological and clinical information of patients diagnosed with LN. The IHC analysis revealed predominant localization of IFI44 expression within the cell in the nucleolus (Supplementary Figure 1), encompassing the glomeruli and tubulointerstitium of patients diagnosed with LN (Figure 2A). Significantly, LN patients showed much higher IFI44 expression in both glomerular and tubulointerstitial regions compared to MCD and NC samples (Figure 2B and C). Compared to patients with classes II, III, and V, there is a pronounced elevation in glomerular IFI44 expression in patients with lupus nephritis classified as Class IV (Figure 2D). Yet, there was no notable variation in tubulointerstitial IFI44 expression among different classifications of LN pathology (Figure 2E). Thus, the variation in glomerular IFI44 expression truly reflects disease progression in LN.

Additionally, lupus nephritis (LN) patients were stratified into two groups based on the mean optical density (MOD) of IFI44 expression measured in their renal tissues. The stratification was applied separately to glomerular and tubulointerstitial compartments. Specifically, the MOD values for the ‘high’ and ‘low’ expression groups were determined as follows: Glomerular Groups (High IFI44 Expression Group: MOD = 1.6396 ± 0.0787, Low IFI44 Expression Group: MOD = 1.0007 ± 0.0361), while in tubulointerstitial Groups (High IFI44 Expression Group: MOD = 6.5198 ± 0.7522, Low IFI44 Expression Group: MOD = 4.7832 ± 0.4852).

These thresholds were established to analyze differences in clinical outcomes and disease severity associated with IFI44 expression levels. Our analysis, as detailed in Supplementary Table 2, revealed significant differences in clinical characteristics between patients with high versus low IFI44 expression. Notably, higher IFI44 expression in both glomerular and tubulointerstitial compartments was associated with more severe disease activity. This correlation underscores the potential of IFI44 as a marker for disease severity in lupus nephritis, suggesting that elevated renal expression of IFI44 could be indicative of an aggressive disease phenotype.

### Colocalization of IFI44 with other indicators among various renal cells in patients diagnosed with LN

**Figure 3.**
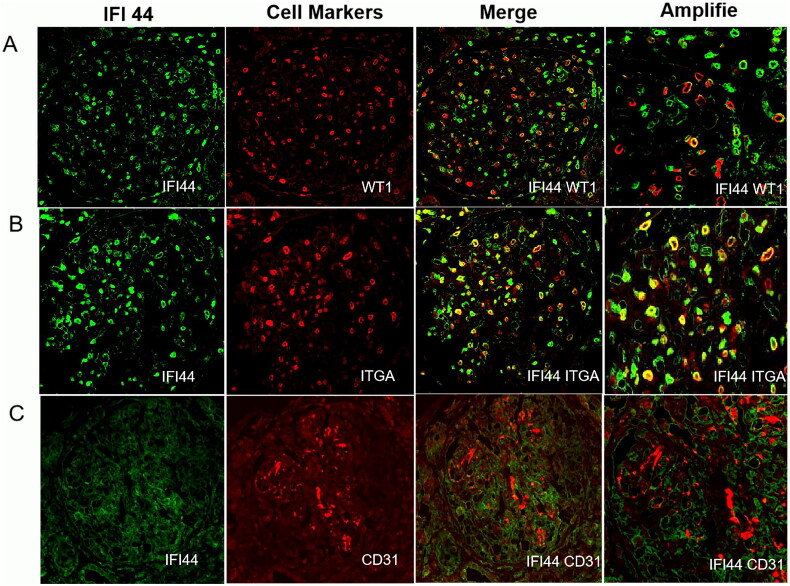
Multiplex immunofluorescence staining for co-localization studies in LN patients. (A) Staining results show that IFI44 (green) colocalizes with WT1 (red), an indicator of podocytes, indicating a potential link between IFI44 expression and podocyte function in LN. (B) It depicts IFI44 (green) co-localizing with ITGA (red), a mesangial cell marker, suggesting IFI44’s involvement with mesangial cells in the disease pathology. However, no co-localization was observed between IFI44 (green) and CD31 (red), an endothelial cell marker.

Multiplex immunofluorescence staining revealed colocalization of IFI44 with other indicators among various renal cells in patients diagnosed with LN. This technique allowed for the detailed exploration of IFI44 expression patterns and its spatial relationships with different populations of renal cells. The analysis revealed that IFI44 co-localizes effectively with both mesangial cells and podocytes in LN patients, indicating that either infiltrating inflammatory cells or renal cells express IFI44 (Figure 3).

### Correlation between IFI44 expression in the kidneys of LN patients and their clinical and pathological characteristics

**Figure 4.**
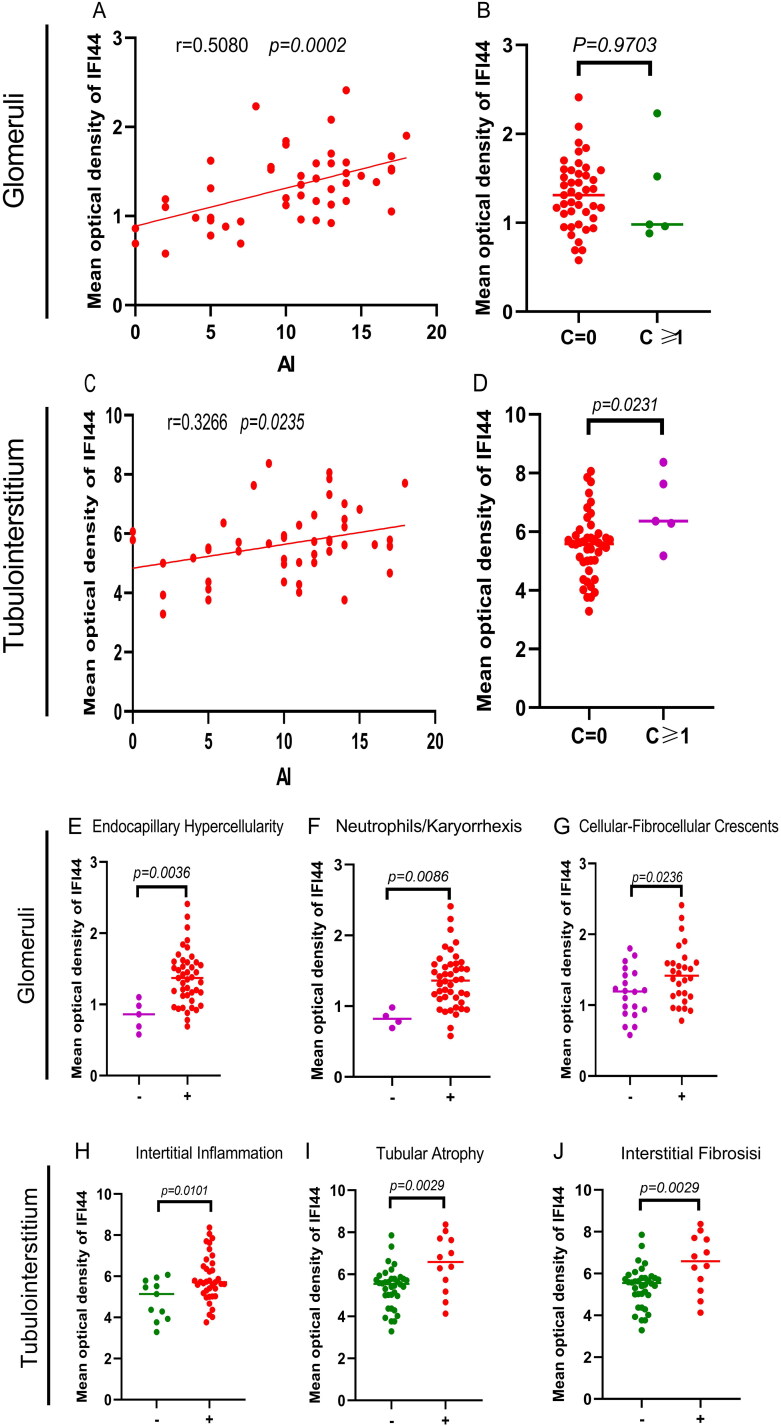
Correlation between pathological indicators and IFI44 expression in the kidneys of LN patients. (A) The correlation of total pathological activity index (AI) with glomerular IFI44 expression is shown in this panel. (B) The total chronicity index (CI) is also associated with glomerular IFI44 expression. (C) It illustrates the connection between tubulointerstitial IFI44 expression and the total AI. (D) It highlights the correlation between tubulointerstitial IFI44 expression and the total CI. Specific pathologic indices associated with IFI44 expression include: (E) Glomerular IFI44 expression and endocapillary hypercellularity. (F) The relationship with neutrophils/karyorrhexis. (G) Association with cellular-fibrocellular crescents. (H) Correlation between interstitial inflammation with tubulointerstitial IFI44 expression. (I) Association with tubular atrophy. (J) Connection to interstitial fibrosis.

To assess the association of IFI44 expression with clinical and pathological characteristics in LN patients, we first investigated its association with the severity of renal damage of LN patients. Glomerular IFI44 expression showed a significant positive correlation with the total pathological activity index (AI) (Figure 4A, *r* = 0.5080, *p* = 0.0002). Glomerular IFI44 expression also correlates with other activity indices, including cellular-fibrocellular crescents, neutrophils/karyorrhexis, and endocapillary hypercellularity (Figure 4E–G), but it does not correlate with the total chronicity index (CI) (Figure 4B).

Similarly, total pathological activity index (AI) has a significant positive correlation with tubulointerstitial IFI44 expression (Figure 4C, *r* = 0.3266, *p* = 0.0235). In LN patients, an increased tubulointerstitial IFI44 expression remarkably correlates with interstitial inflammation (Figure 4H, *p* = 0.0101). Moreover, in LN patients with renal chronicity (CI1), an increased tubulointerstitial IFI44 expression was detected related to patients with lower IFI44 expression levels (Figure 4D, CI = 0). In parallel, in patients with either tubular atrophy or interstitial fibrosis, we observed an elevated tubulointerstitial IFI44 expression.

In LN patients, Figure 5A–D outlines the relationships between IFI44 expression and clinical indices in their kidneys. Glomerular IFI44 expression was significantly positively correlated with both serum levels of creatinine and scores of SLEDAI. Conversely, glomerular IFI44 expression was significantly negatively correlated with eGFR and the levels of baseline complement C3. As shown in Figure 5G, SLEDAI scores also positively correlate with tubulointerstitial IFI44 expression.

Figure 5.Correlation of renal IFI44 expression with LN patients’ clinical characteristics.This figure displays the associations between IFI44 expression in renal regions and various clinical characteristics:Correlation of glomerular IFI44 expression with (A) SLEDAI scores (r = 0.5146, P = 0.0002), (B) serum creatinine levels (r = 0.3246, P = 0.0244), (C) serum C3 levels (r = -0.3052, P = 0.0349), (D) eGFR (r = -0.3934, P = 0.0057), (E) serum C4 and (F) hematuria.Correlation of interstitial IFI44 expression with (G) SLEDAI scores (r = 0.4152, P = 0.0033), (H) serum creatinine levels, (I) eGFR, (J) serum C3, (K) serum C4, and (L) hematuria.

**Figure:**
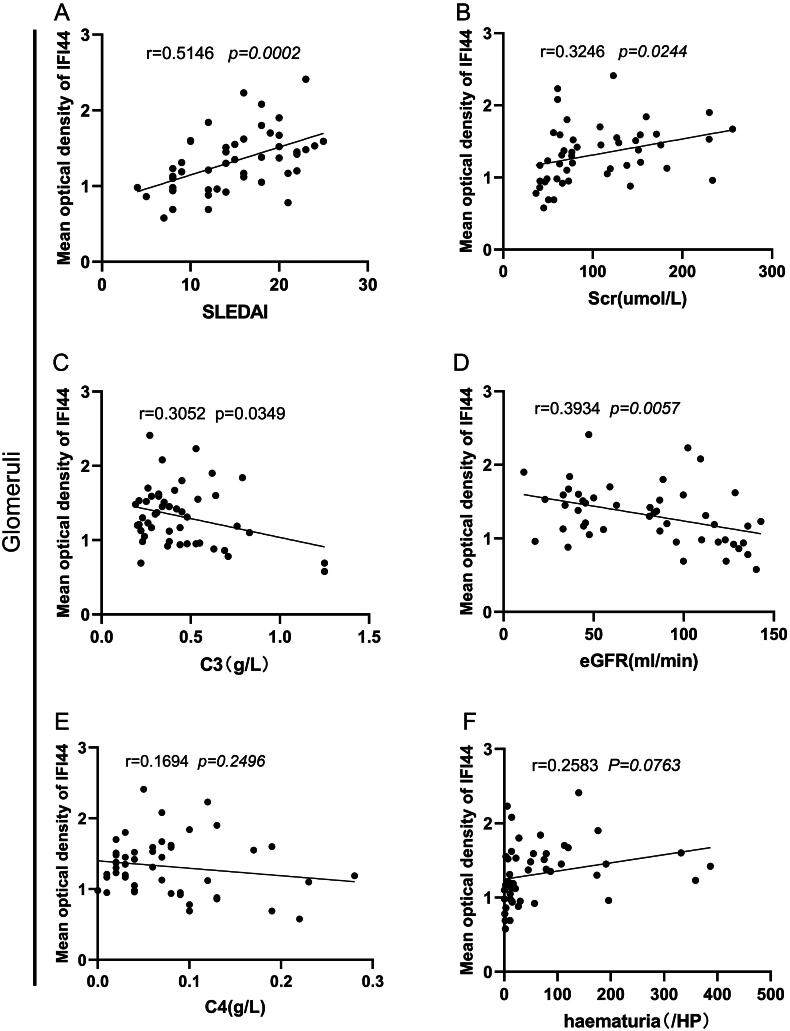

**Figure:**
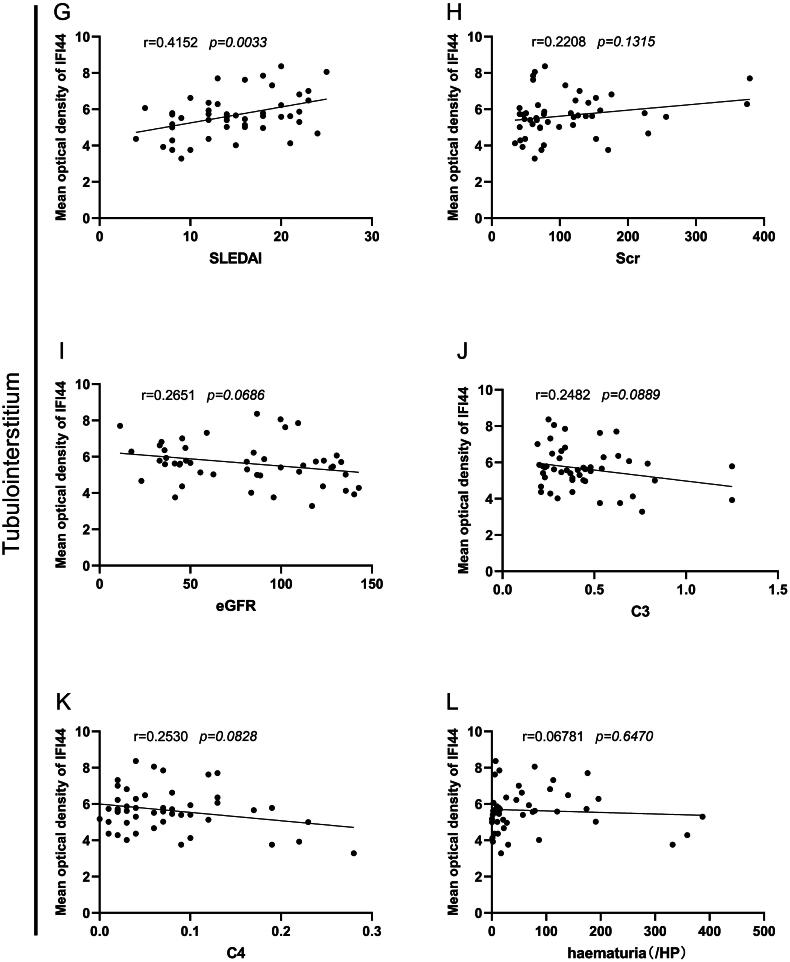

Based on the average optical density (AOD) of IFI44 expression in LN patients, two groups were stratified, facilitating a comprehensive analysis of the biomarker’s impact on disease progression and treatment outcomes.

### GSEA: the role of IFI44 in the process of LN development

**Figure 6.**
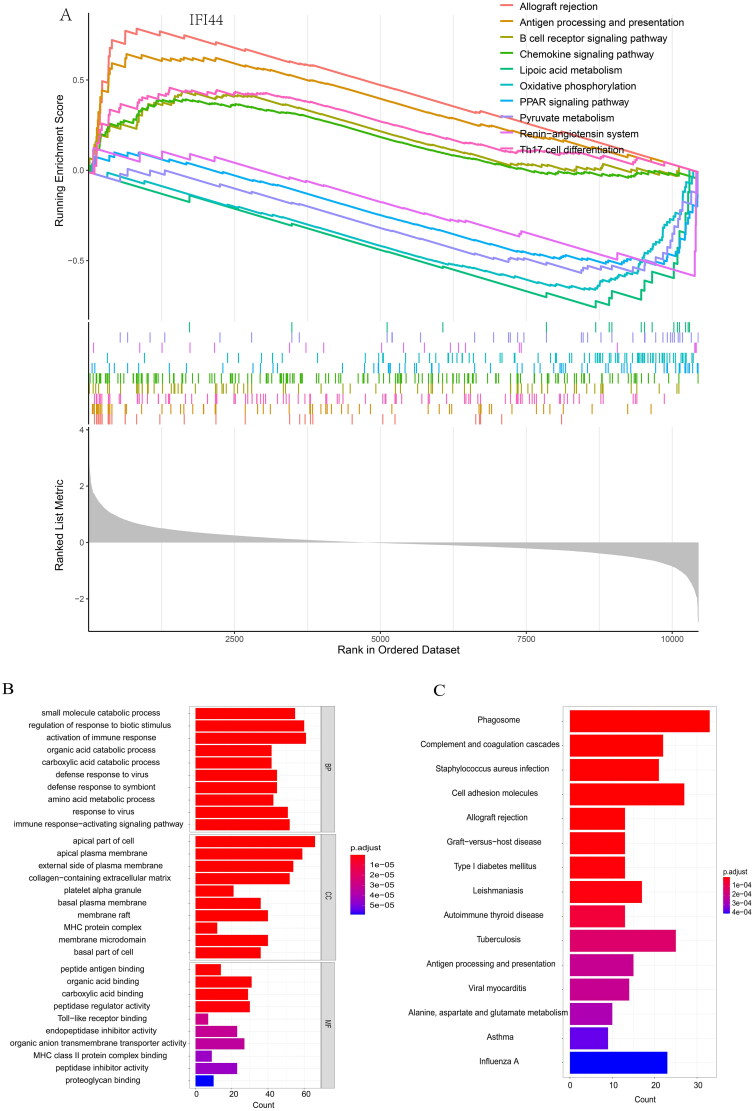
Biological processes and mechanisms associated with expression of IFI44. The involvement of IFI44 in body biological processes and their potential mechanisms are shown in this figure. A. GSEA data on the identification of key biological processes related to variations in IFI44 expression. B & C. Analysis results of GO and KEGG on the association of IFI44 levels with various biological processes.

In our investigation into how IFI44 affects LN progression, we employed GSEA analysis, which highlighted several pathways significantly boosted in patients with higher IFI44 expression, including chemoattractant signaling, signaling pathways associated with B cell receptor, allograft rejection, processing and presentation process associated with antigen and helper T cell 17 differentiation. Conversely, there was a notable reduction in the PPAR signaling pathway, pyruvate metabolism, renin-angiotensin system, oxidative phosphorylation, and fatty acid metabolism (Figure 6A).

GO-BP enrichment analysis focused primarily on small molecule catabolism, regulation of responses to bio-stimuli, and activation of immune responses.
2. GO-CC analysis indicated that corresponding genes are intensified in the apical plasma membrane, apical region of the cell, and outer plasma membrane.
GO-MF analysis revealed enrichment in pathways related to peptidase-regulated activity, binding process of peptide antigen, Toll-like receptor, and MHC class II protein complex (Figure 6B).
KEGG pathway analysis suggested associations with several pathways related to immune functions: allograft rejection, graft-versus-host disease, antigen processing and presentation, cell adhesion molecules, as well as complement and coagulation cascades (Figure 6C).

Collectively, the findings from GSEA, GO, and KEGG analyses imply that the development of LN may be promoted by immune-related processes related to IFI144. Based on these insights, we proceeded with immunological analyses.

### IFI44 as a factor involved in immunological functions contributing to the development of LN

Due to the critical involvement of immune processes in LN development, we utilized the CIBERSORT algorithm to explore the characteristics of immune cells in LN patients compared to healthy individuals. The analysis revealed that in LN patients, there are an increase in several immune cells, including resting mast cells, M2-macrophages, and γδ-type T cells. While other types of immune cells such as CD8+ T cells, follicular helper T cells, and activated mast cells are less prevalent.

**Figure 7.**
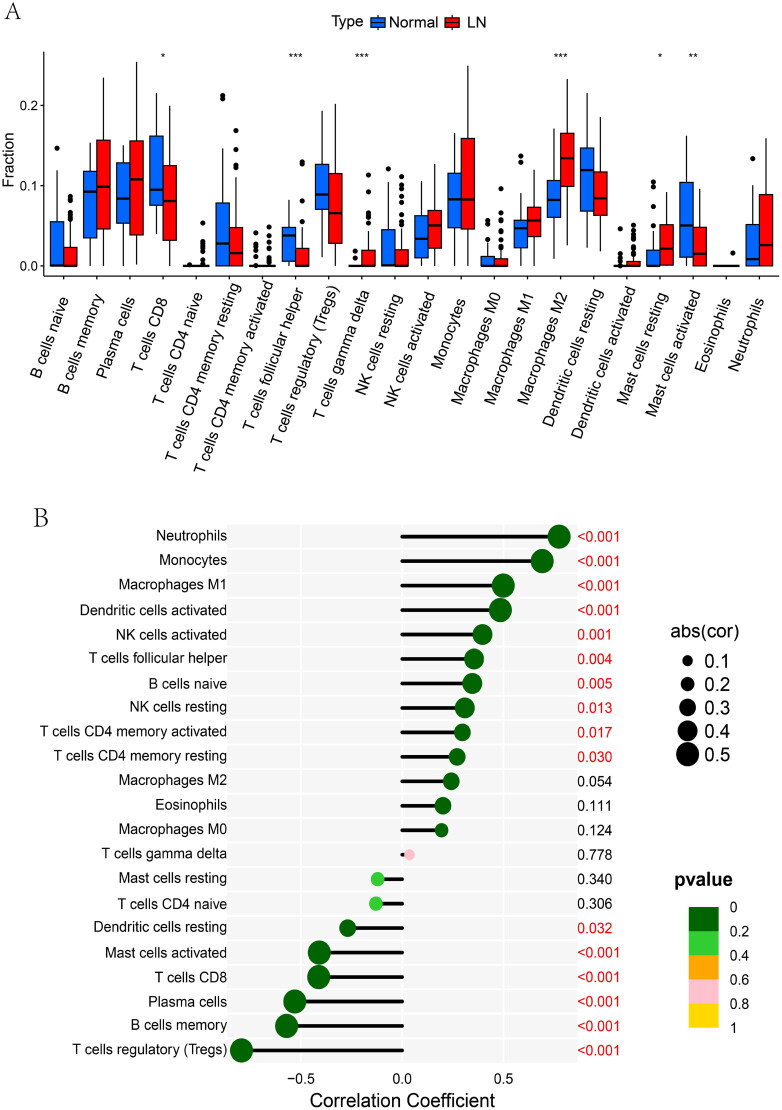
Immune cell infiltration analysis. The differential pattern of infiltrated immune cells in the kidneys of LN patients compared to healthy individuals was identified using bioinformatics tools like the CIBERSORT algorithm. A. A violin plot depicts a distinct infiltration pattern of immune cells in LN patients. B. The association of IFI44 with various infiltration patterns in subpopulations of immune cells was also examined.

Notably, IFI44 expression showed significant correlations with various immune cells. There are a statistically significant positively correlation of IFI44 with the following immune cells: neutrophils, M1 macrophages monocytes, activated dendritic cells, follicular helper T cells, initial B cells, resting natural killer cells, activated memory CD4+ T cells, and resting CD4+ memory T cells (Figure 7A).

There are also a statistically significant negatively correlation of IFI44 with the following immune cells: regulatory T cells, plasma cells memory B cells, CD8+ T cells, activated mast cells, and resting dendritic cells. Our study revealed that IFI44 as a factor involved in immunological functions contributing to the development of LN (Figure 7B).

## Discussion

Since LN significantly contributes to the incidence and mortality of SLE, and due to the lack of effective pharmacological treatments, LN patients often progress to chronic kidney disease (CKD) or end-stage renal disease (ESRD) [16,17]. Although recent advances have improved the management of LN-related morbidity and mortality, flare-ups remain prevalent, and achieving remission is difficult even with optimal therapy [18,19]. Therefore, identifying biomarkers and potential therapeutic targets is crucial for improving the prognosis and reducing mortality rates among LN patients.

Recently, the integration of bioinformatics and machine learning algorithms has been pivotal in uncovering useful indicators in disease diagnose. Our research explored a promising indicator (IFI44) for LN diagnose through these advanced techniques. Upon our knowledge, we are the first research group using the immunohistochemistry (IHC) to examine IFI44 expression in the LN patients’ kidneys (*n* = 48). The expression level of IFI44 in the kidneys of LN patients was significantly up-regulated and closely correlated with the extent of kidney damage compared to normal individuals.

It is noteworthy that expression of IFI44 in the glomerular region closely correlates with indicators of active renal pathology. On the other hand, tubulointerstitial IFI44 expression was linked to markers of chronic renal pathology. Additionally, higher IFI44 levels were correlated with increased SLEDAI scores, serum creatinine, hematuria, eGFR levels, and serum complement C3. Our research highlights the clinical value of renal biopsy to predict clinical outcomes in LN cases, identifying IFI44 as a significant biomarker for diagnosis and prognosis.

IFI44 is a protein induced by interferon (IFN) and was initially identified during the isolation of microtubule-aggregating proteins associated with hepatitis C virus [20]. IFNs are a diverse group of cytokines, and over five decades of research have established their critical role as major regulators of the immune system in human being, significantly impacting autoimmune diseases [21,22]. IFN has also been identified as a major contributing factor in LN [23]. Previous research has demonstrated that serum IFI44 levels can effectively differentiate LN patients from healthy individuals [24]. Similarly, our investigation revealed that IFI44 expression in the kidneys can differentiate LN patients from healthy controls, with a notable increase in IFI44 gene expression observed in LN patients’ kidneys.

The subcellular localization of IFI44 has been subject to varying observations in previous studies, which may reflect its diverse roles in cellular physiology and pathology. Hallen et al. [25] noted that when IFI44 is exogenously expressed, it predominantly localizes to the cytoplasm. Conversely, Power et al. [26] reported nuclear localization of IFI44 in HEK293 cells following HIV-1 infection, suggesting a context-dependent redistribution. In our study, IFI44 expression in lupus nephritis (LN) patients predominantly localized to the nucleus, contrasting with its distribution in both the nucleus and cytoplasm in Minimal Change Disease (MCD) and normal controls (NC). This differential localization might be indicative of IFI44’s involvement in specific cellular pathways or functions that are activated under pathological conditions such as LN. The nuclear presence of IFI44 in LN could be associated with specific gene regulation processes that are triggered during autoimmune responses. Understanding the factors that dictate IFI44’s localization and function requires further investigation and could uncover novel insights into its role in the pathogenesis of LN and other diseases. Such studies could potentially reveal new therapeutic targets or diagnostic markers, emphasizing the need for future research in this direction. Significantly, IFI44, when used as a specific biomarker for LN, demonstrated the ability to differentiate the active LN patients from those with inactive LN. In the literature, LN patients classified with higher pathological stages (III, IV, or V) are at increased risk of developing chronic kidney disease due to irreversible renal damage, thus reducing renal lifespan [27]. This research demonstrated that in LN patients with higher pathological stages, renal IFI44 expression is significantly up-regulated, particularly in those classified as class IV. These findings align with research conducted by Lingling Shen [10], suggesting that IFI44 biomarker may have the potential to predict LN outcomes. While previous studies, including the work by Lingling Shen et al. have explored the role of IFI44 as a potential biomarker in lupus nephritis, our research extends these findings by specifically correlating IFI44 expression with disease severity through detailed immunohistochemical analysis. Unlike earlier studies that may have focused primarily on the presence and basic patterns of IFI44 expression, we have conducted an in-depth analysis that examines how the specific expression sites of IFI44 within kidney tissue correlate with clinical pathology. This approach allows us to not only confirm IFI44’s presence but also to evaluate its differential expression in various kidney compartments and its direct association with the pathological features of lupus nephritis.

By linking IFI44 expression to disease severity, our study provides critical insights that could influence the clinical management of lupus nephritis. Understanding the localization and intensity of IFI44 expression offers a more nuanced view of its role in the disease process, potentially guiding more targeted therapeutic interventions. This detailed analysis helps to better assess disease severity and could lead to improved personalized treatment strategies, thereby enhancing patient outcomes in lupus nephritis.

Furthermore, IFI44 could be considered a novel target for therapy aimed at inhibiting type I IFN production in LN patients [28], suggesting the dual function of IFI44 in LN diagnosis and treatment.

This research provides fresh perspectives on the trajectory and prognosis of lupus nephritis (LN). Previous research has shown that genes associated with LN are involved mainly in immune and inflammatory processes [29,30]. These findings, however, lacked validation in the specific renal expressions seen in LN patients, prompting our more exhaustive study. We explored the underlying mechanisms and the cellular makeup influencing LN. GSEA analysis disclosed that higher IFI44 expression is prevalent in immune reactions. Additionally, results from the CIBERSORT algorithm revealed that IFI44 expression is significantly associated with essential genes and infiltrating monocytes and macrophages in kidneys of LN patients. Specifically, monocytes were the primary cell type in LN glomeruli, and M2 macrophages were chiefly found in the tubular interstitium, both correlating positively with critical gene expression, in line with prior research [31]. Monocytes are pivotal in either innate or adaptive immune responses [32], involved in the process of inflammation linked to LN glomerulopathy [33]. Conversely, M2 macrophages are involved in renal fibrosis development [34–37], suggesting the potential roles of an increased activity of key genes in severe kidney damage in LN patients, which aligns with our study’s outcomes. Importantly, our results also showed the co-localization of IFI44 with podocytes and tethered cells in LN renal tissues, highlighting IFI44’s involvement in LN’s renal immune response and its potential contribution to disease progression through various pathways.

We recognize the limitations inherent to our study, as it is a single-center retrospective analysis. Future directions include conducting multicenter, prospective studies and gathering extensive prognostic data to deepen our understanding of IFI44’s impact on LN.

## Conclusion

Renal IFI44 expression in LN patients holds promise as a valuable indicator for assessing the extent of kidney damage and predicting prognosis. Leveraging renal IFI44 expression could enhance the precision in predicting renal responses and tailoring personalized treatment strategies for LN.

## Supplementary Material

Supplementary Figure 1.jpg

Figures and tables.docx

## Data Availability

The datasets supporting the conclusions of this study are publicly available in the Gene Expression Omnibus (GEO) database.
