# Supplementary figures and images for: Determining IFI44 as a key lupus nephritis’s biomarker through bioinformatics and immunohistochemistry
Source: Ren Fail. 2025 Mar 18;47(1):2479575. doi: 10.1080/0886022X.2025.2479575 (PMC11921169; doi:10.1080/0886022X.2025.2479575)

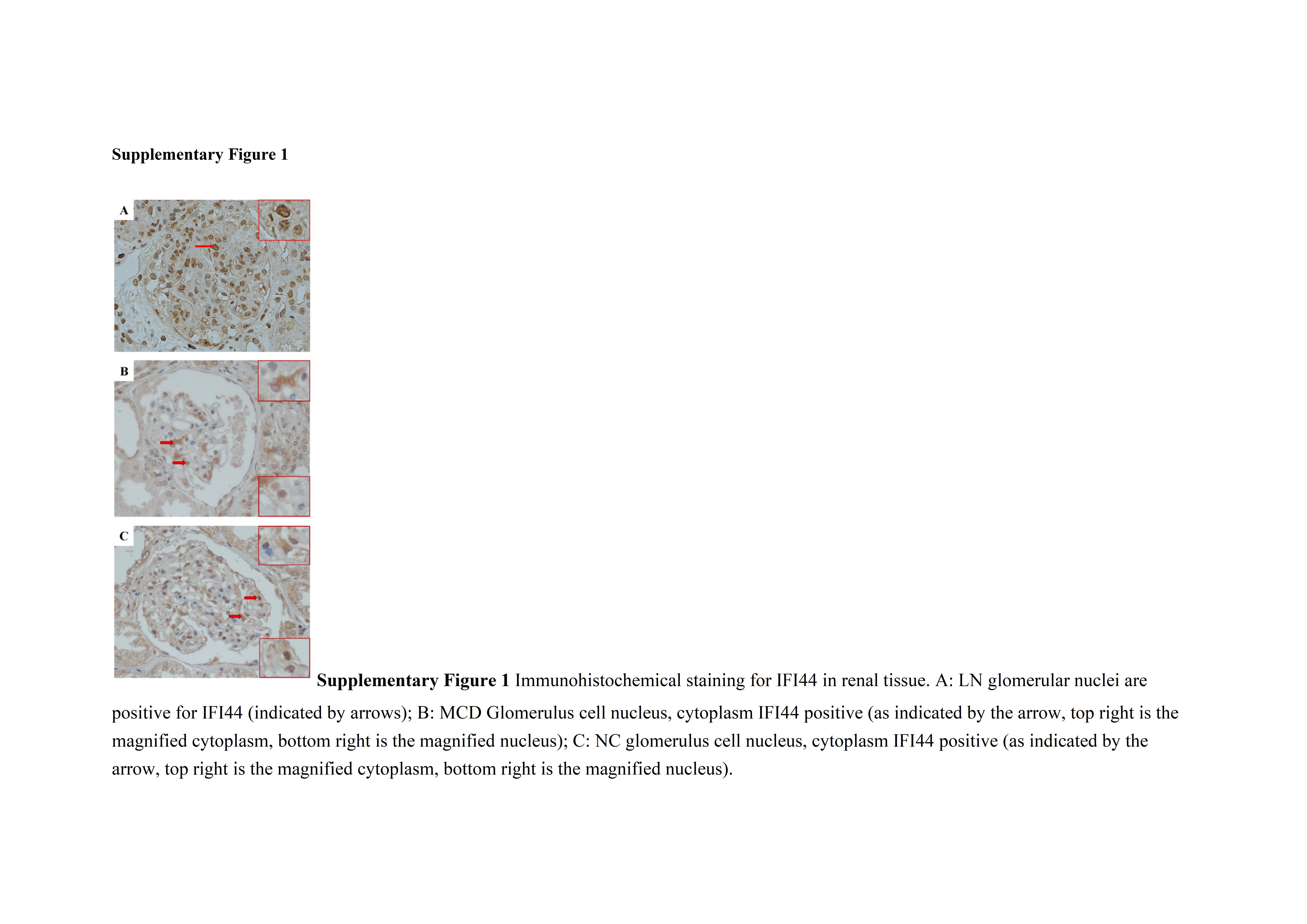

Supplement: Supplementary Figure 1.jpg [file IRNF_A_2479575_SM2184.jpg]
