# Supplementary material for: Determining IFI44 as a key lupus nephritis’s biomarker through bioinformatics and immunohistochemistry
Source: Ren Fail. 2025 Mar 18;47(1):2479575. doi: 10.1080/0886022X.2025.2479575 (PMC11921169; doi:10.1080/0886022X.2025.2479575)
Supplement: Figures and tables.docx [file IRNF_A_2479575_SM2183.docx]

**Fig. 1 Identification of critical indicators of lupus nephritis (LN)**


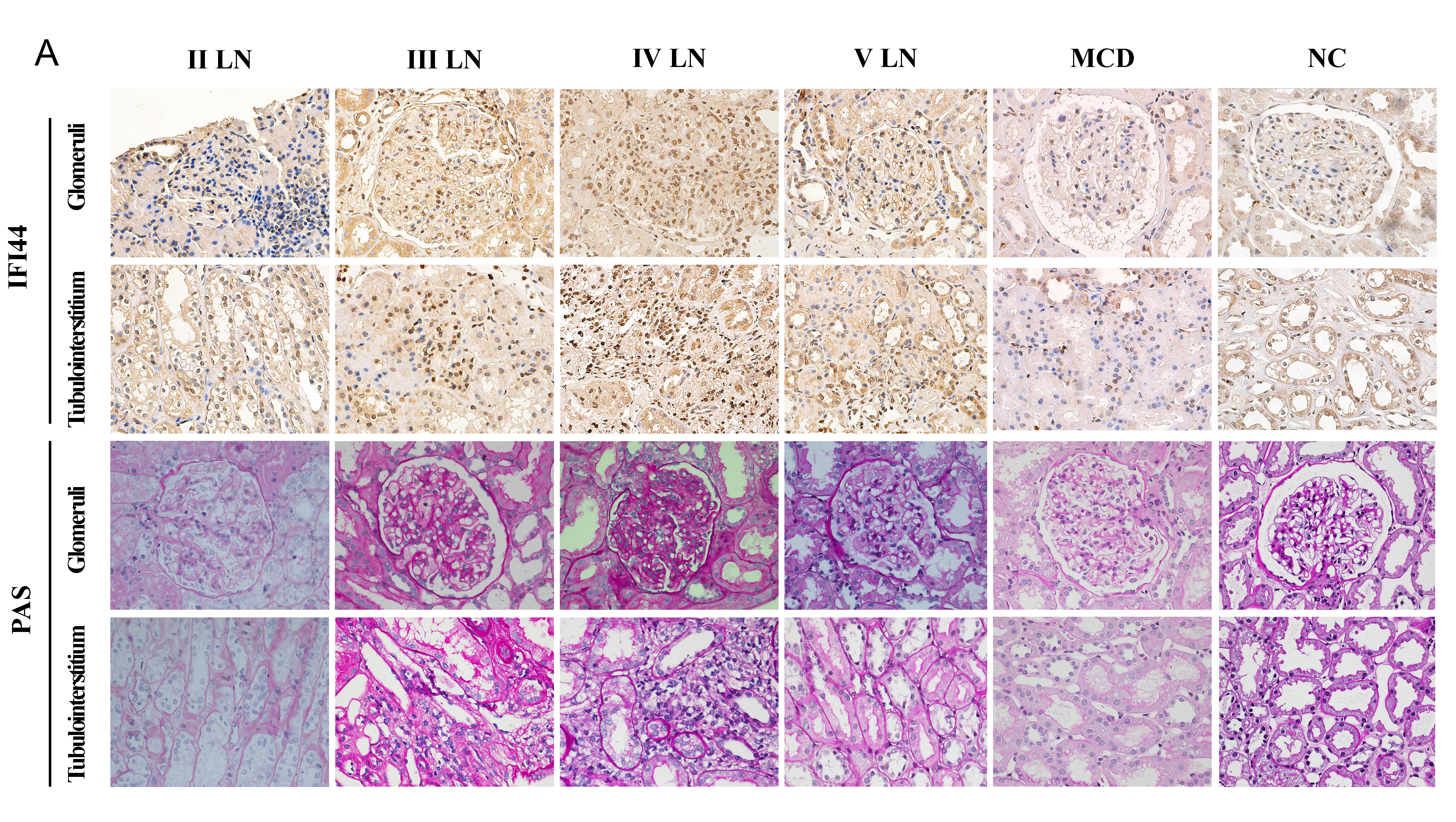


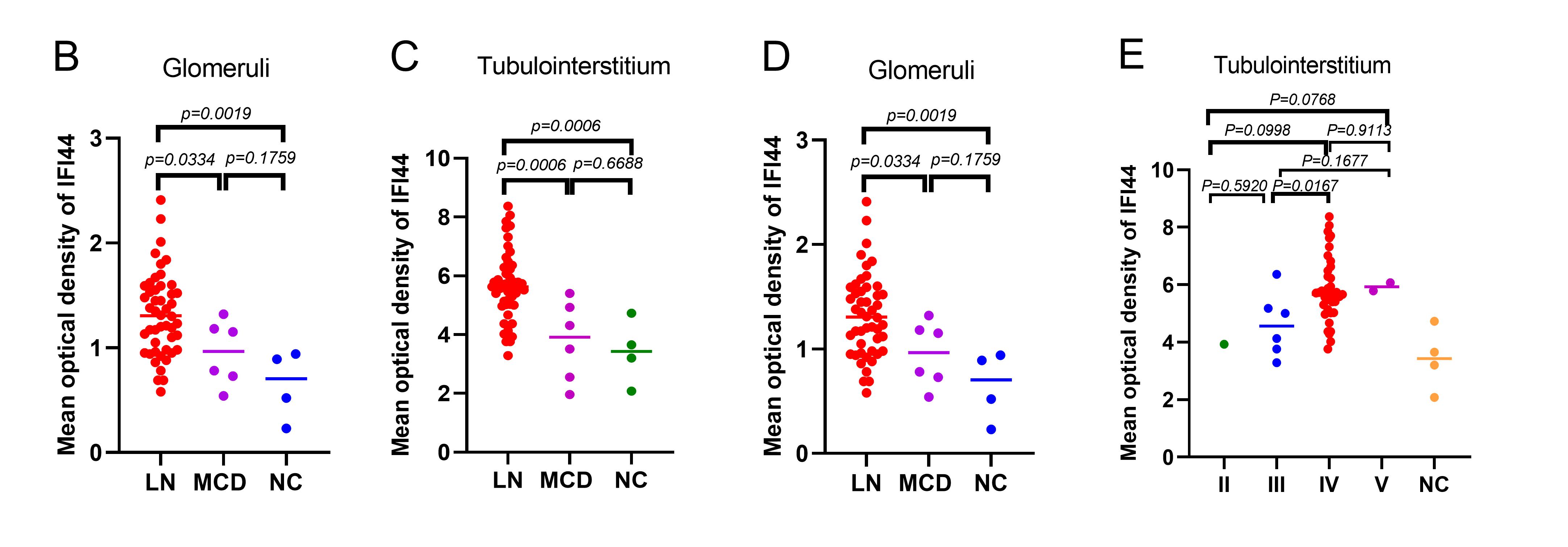


**Fig. 2 Immunohistochemical results of IFI44 expression in renal tissues**


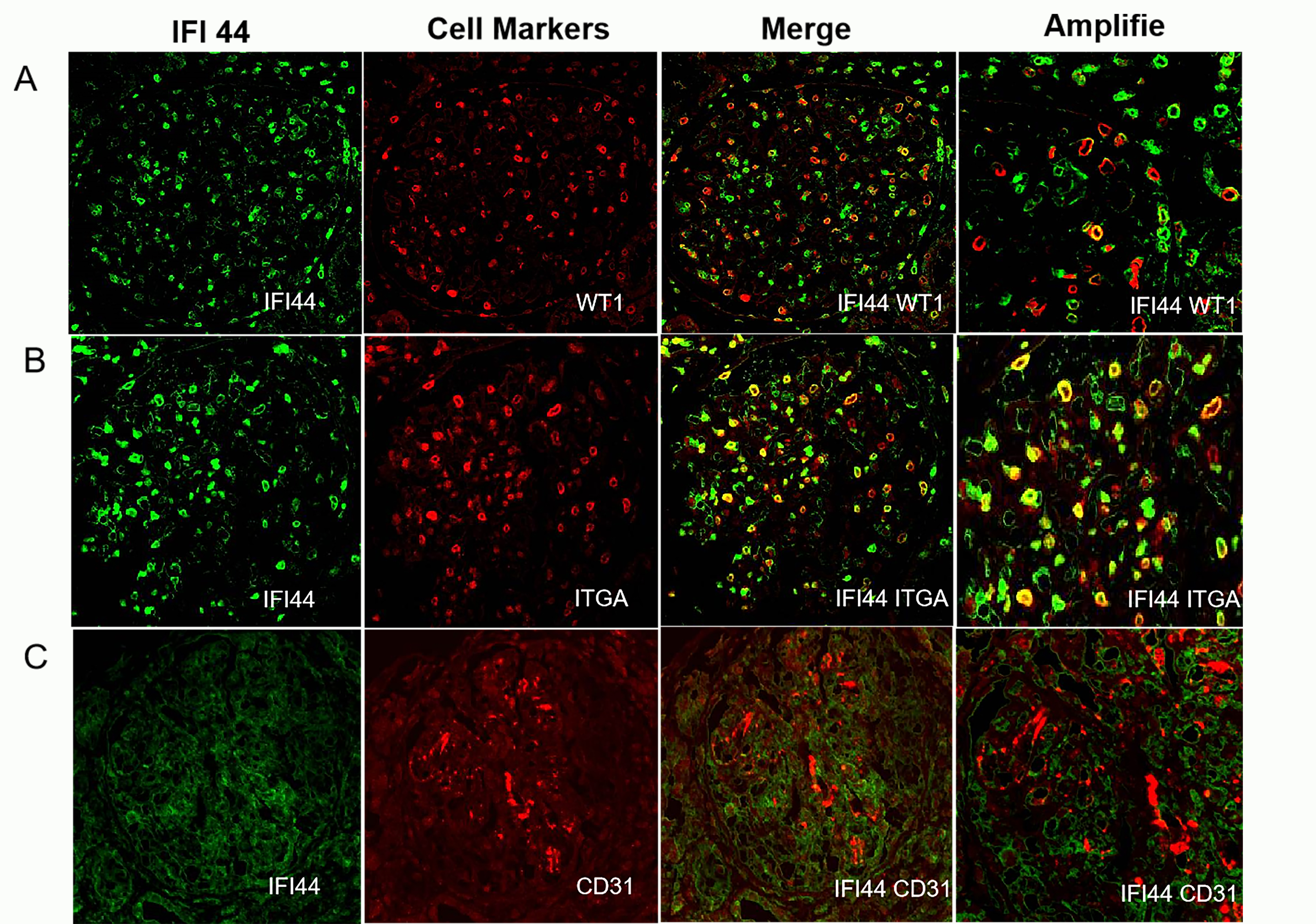


**Fig. 3 Multiplex immunofluorescence staining for co-localization studies in LN patients**

**
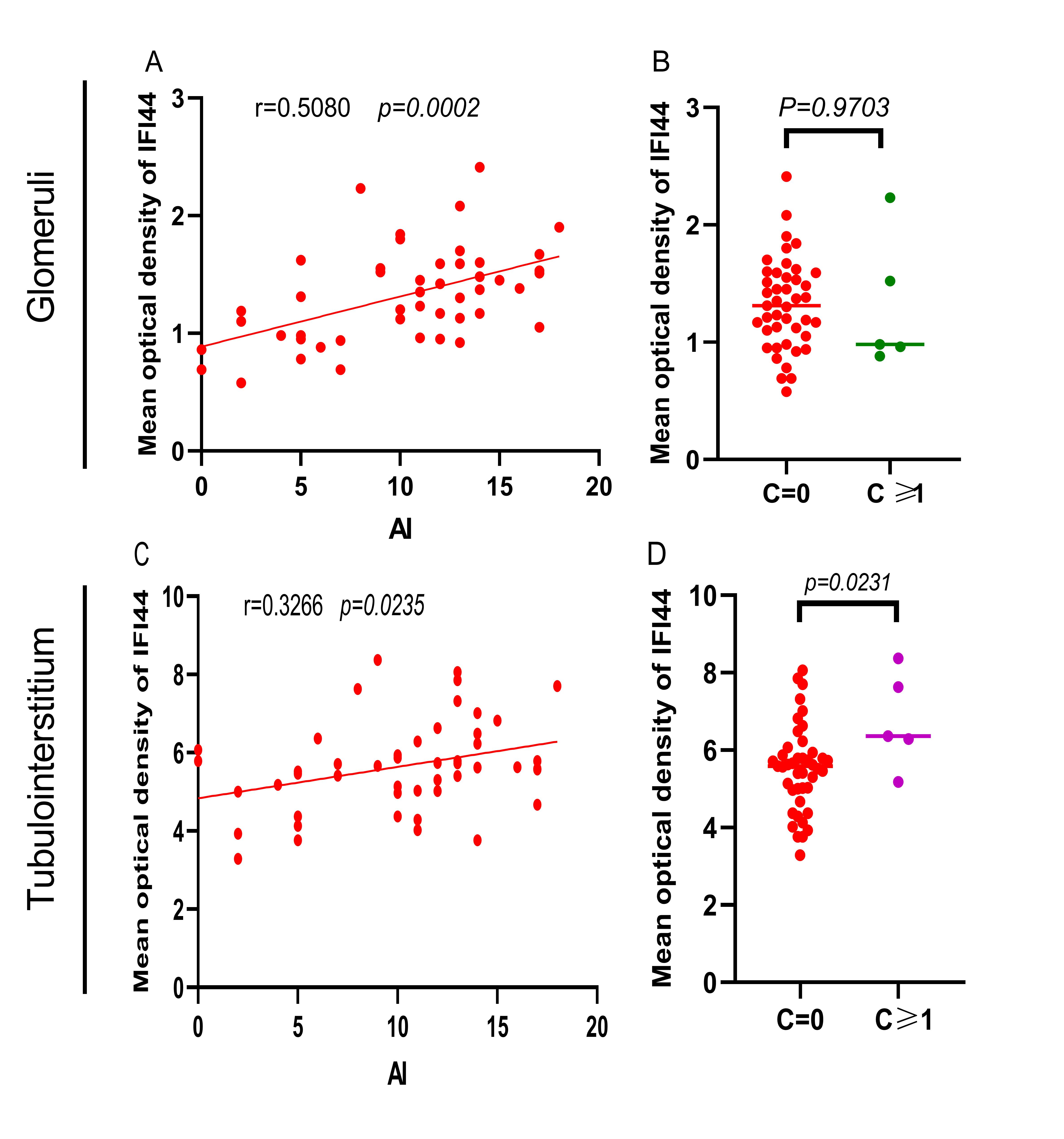
**

**
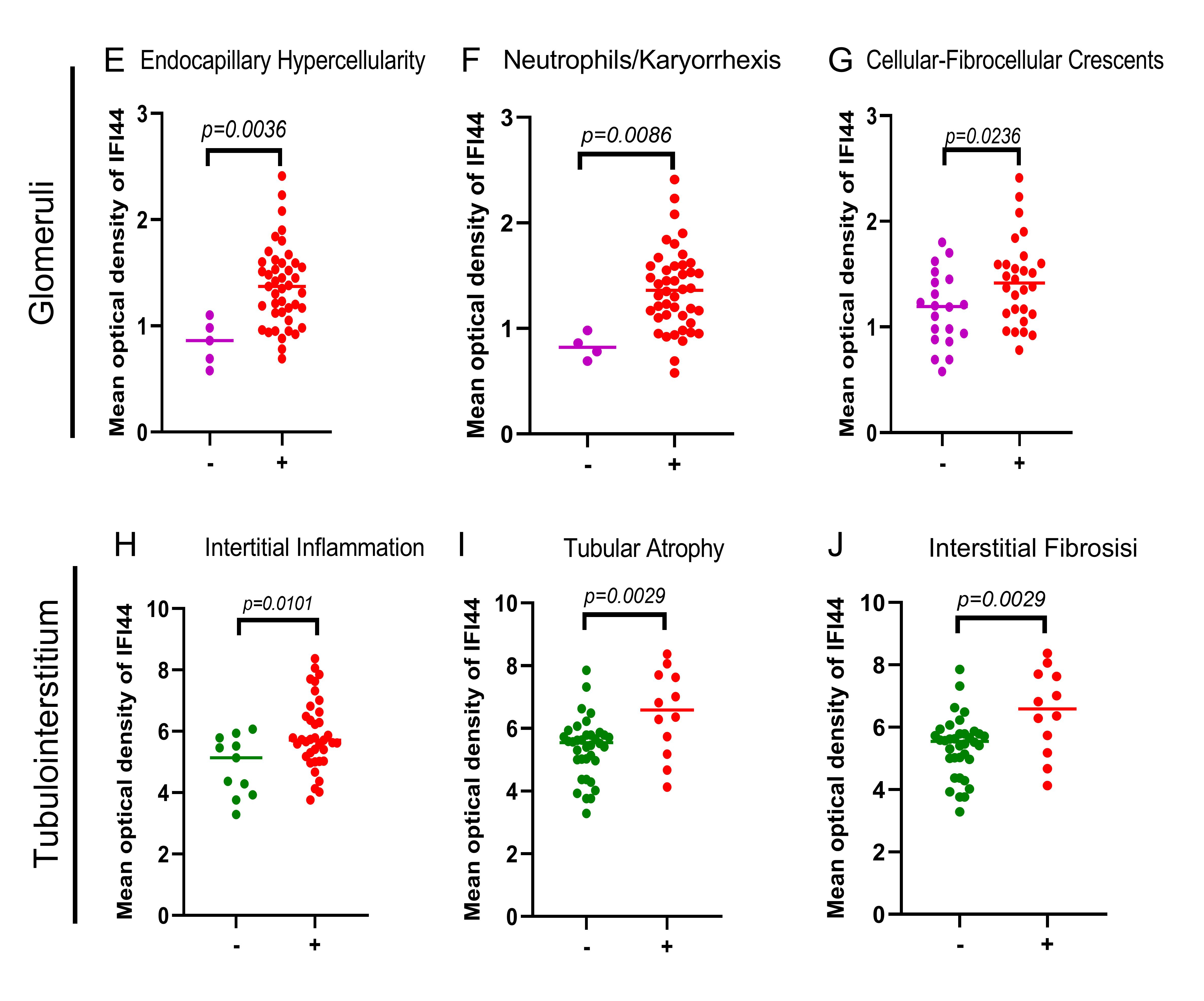
**

**Fig. 4** **Correlation between pathological indicators and IFI44 expression in the kidneys of LN patients**


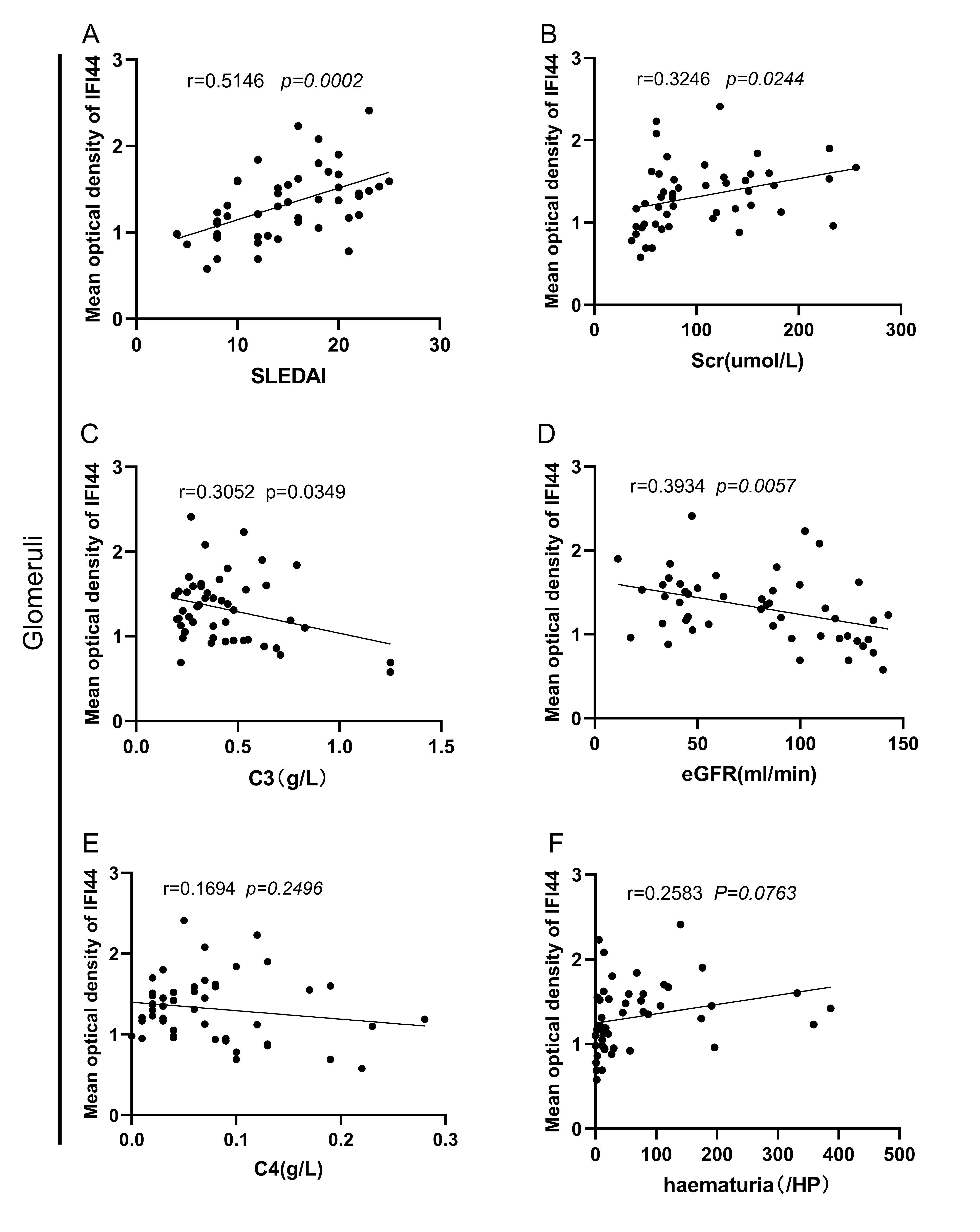


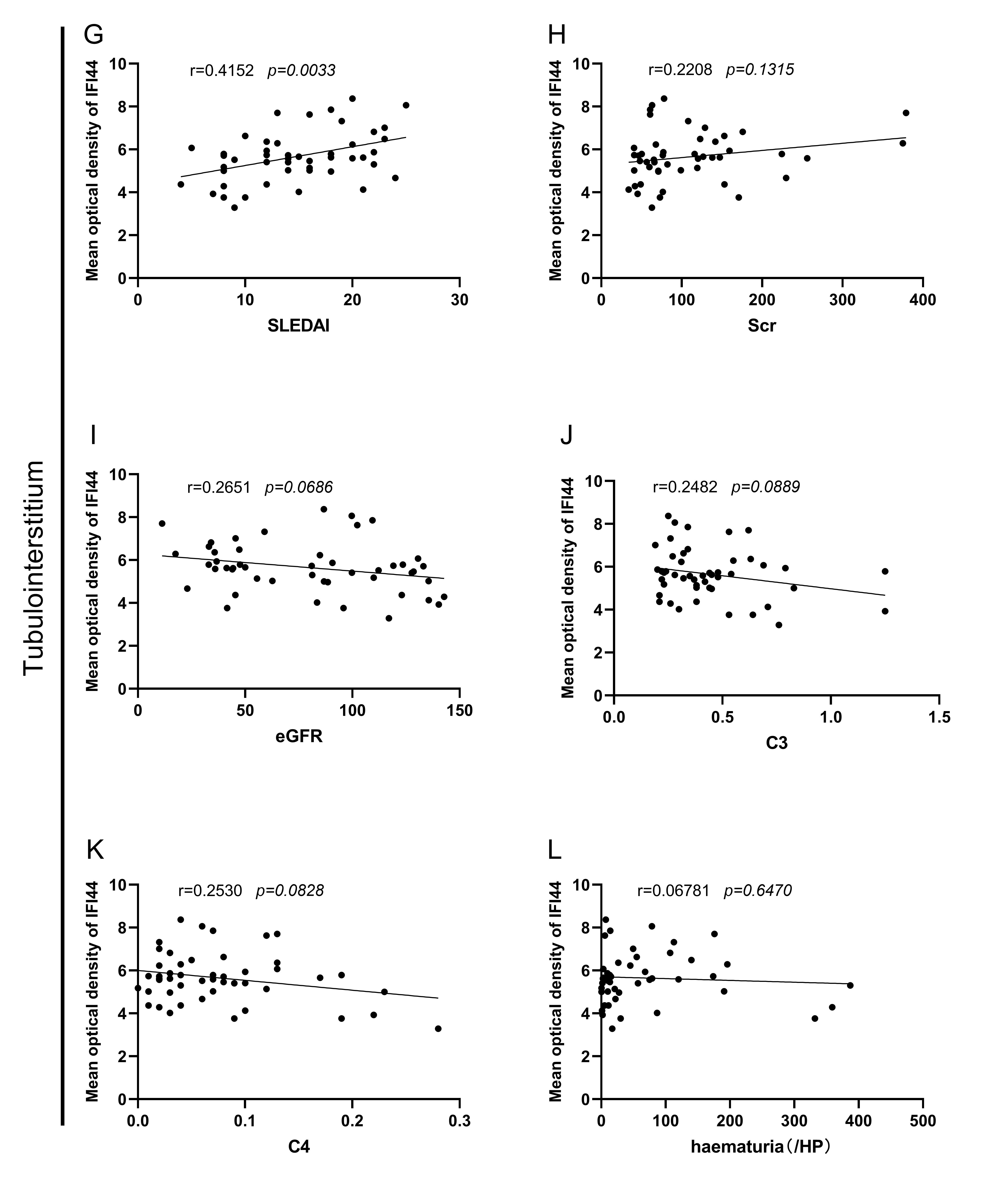


**Fig. 5 Correlation of renal IFI44 expression with LN patients’ clinical characteristics**


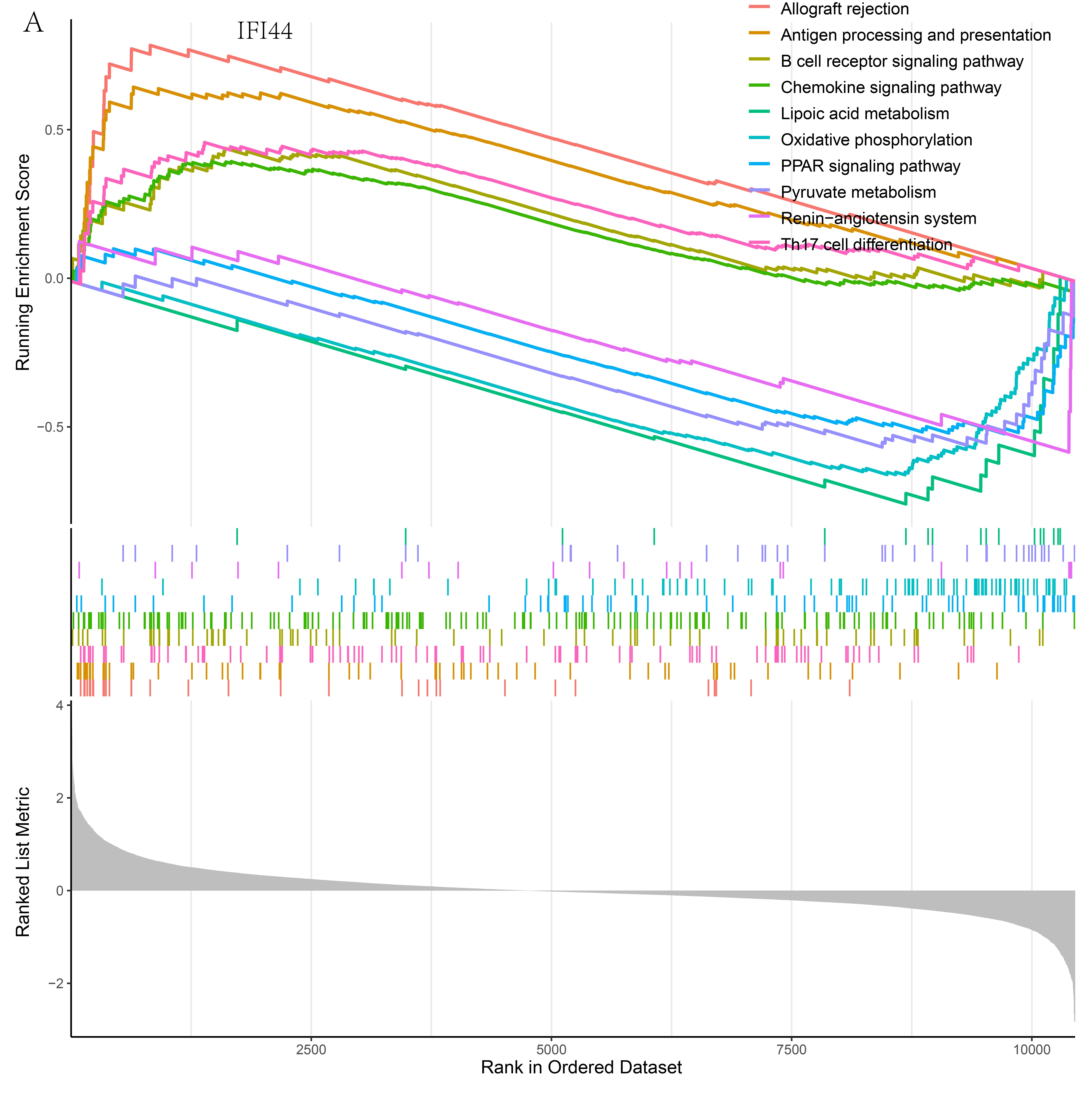





**Fig. 6 Biological processes and mechanisms associated with expression of IFI44**

**
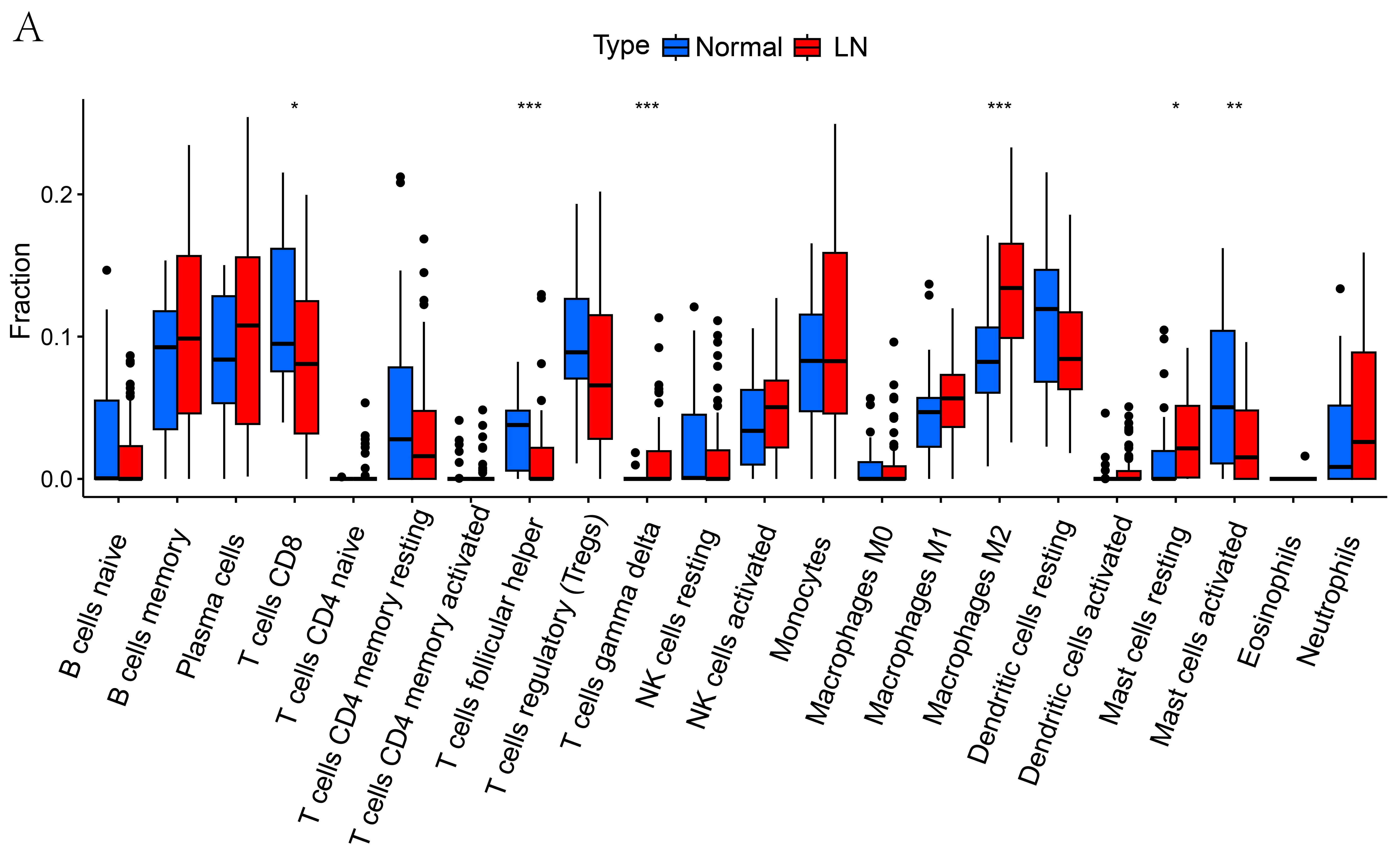
**

**
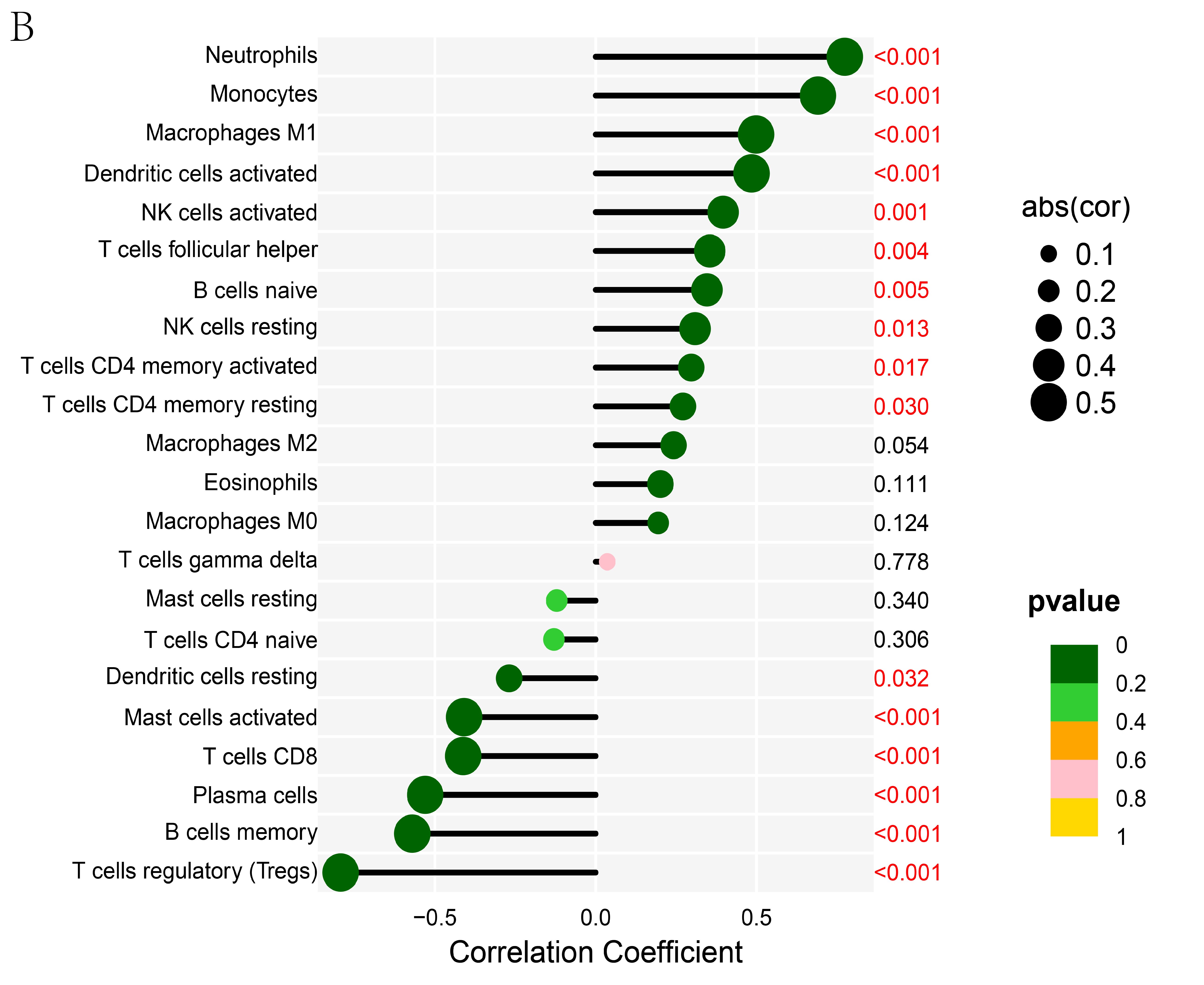
**

## **Fig. 7 Immune cell infiltration analysis**

**Supplementary Figure 1**


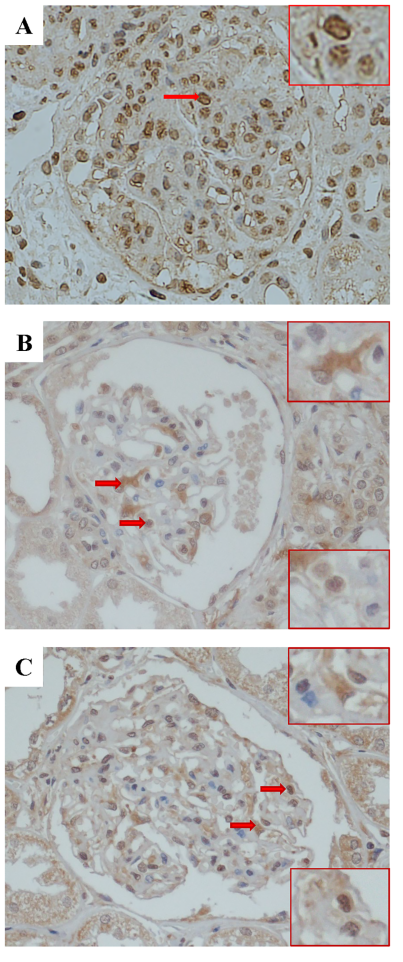
**Supplementary Figure 1** Immunohistochemical staining for IFI44 in renal tissue. A: LN glomerular nuclei are positive for IFI44 (indicated by arrows); B: MCD Glomerulus cell nucleus, cytoplasm IFI44 positive (as indicated by the arrow, top right is the magnified cytoplasm, bottom right is the magnified nucleus); C: NC glomerulus cell nucleus, cytoplasm IFI44 positive (as indicated by the arrow, top right is the magnified cytoplasm, bottom right is the magnified nucleus).

**Supplemental Table 1: General clinical and pathological data of lupus nephritis patients**

| **Clinical information** | | **Laboratory Assessment** | | **Renal Histopathology** | |
| --- | --- | --- | --- | --- | --- |
| Sex (male/female), no. | 13/91 | Hematuria, no. (%) | 41（85.42） | Classification |  |
| Age (years, mean+SD) | 35.38±11.64 | Leukocyturia(noninfectious), no. (%) | 38（79.1） | II, no. (%) | 1(2.08) |
| Hypertension, no. (%) ^a^ | 23(47.91) | Proteinuria(g/24h), mean±SD | 6.28±4.26 | III, no. (%) | 6(12.5) |
| Renal function impaired, no. (%); | 15（31.25） | Serum creatinine (µmol/L), median (range) | 76.95（36.5-256） | IV, no. (%) | 39(81.25) |
| Nephrotic syndrome, no. (%) | 32(66.67) | Low serum C3, no. (%) | 46（95.83） | V, no. (%) | 2(4.17) |
| SLEDAI (mean+SD) | 14.73±5.56 | Low serum C4, no. (%) | 38（79.1） | Activity indices score, median (range) | 11（0-18） |
|  |  | ANA (+), no. (%) | 48（100） | Endocapillary hypercellularity (+), no. (%) | 43(89.58) |
|  |  |  |  | Cellular-Fibrocellular crescents, (+), no. (%) | 28(58.33) |
|  |  |  |  | Neutrophils/Karyorrhexis (+), no. (%) | 44(91.67) |
|  |  |  |  | Interstitial inflammatory cell infiltration, no. (%) | 37（77.08） |
|  |  |  |  | Chronicity indices score, median (range) | 0（0-6） |
|  |  |  |  | Glomerular sclerosis, (+), no. (%) | 12（25） |

SLEDAI: systemic lupus erythematosus disease activity index. ANA: antinuclear antibodies. Anti-dsDNA: anti-double-stranded DNA antibody. mean+SD: mean+ standard deviation. a: Hypertension: blood pressure > 140/90 mmHg or the use of anti-hypertensive agents.

**Supplemental Table 2: Comparison of clinical characteristics of LN patients with high and low IFI44 expression**

| Clinical items Overall | | **Glomeruli** | | | | | **Tubulointerstitium** | | |
| --- | --- | --- | --- | --- | --- | --- | --- | --- | --- |
|  |  | High expression | | Low expression | | *P* | High expression | Low expression | *P* |
| Number | 48 | | 24 | | 24 | - | 24 | 24 | - |
| Male.no. (%) | 3(12.5) | | 4（16.67） | | 0.683 | 0.236 | 2（8.33） | 5（20.83） | 0.220 |
| Age (yrs), mean ± SD | 38.83±9.49854 | | 34.08±10.99 | | 0.075 | 0.799 | 37.75±10.41 | 35.17±10.52 | 0.423 |
| Serum creatinine (µmol/L), median (range) | 122.00（56-256） | | 84.50（36.5-233.7） | | 0.006 | 0.008 | 112（40.8-233.7） | 71.05（36.5-256） | 0.374 |
| C3 (g/L), mean ± SD | 0.39±0.14 | | 0.49±0.30 | | 0.131 | 0.003 | 0.43±0.24 | 0.45±0.24 | 0.807 |
| Proteinuria (g/24 h), mean ± SD | 6.28±4.26 | | 5.93±4.87 | | 6.63±3.53 | 0.570 | 5.4±4.34 | 4.36±4.13 | 0.538 |
| SLEDAI, mean ± SD | 14.73±5.56 | | 17.63±4.49 | | 11.83±4.99 | 0.001 | 17±5.52 | 14±5.36 | 0.144 |
| Hypertension, no. (%) | 24(50) | | 13（54.17） | | 11（45.83） | 0.564 | 14（58.33） | 10(41.67) | 0.248 |
| Renal function impaired, no. (%) | 13（27.08） | | 8（33.33） | | 5（10.42） | 0.329 | 8（33.33） | 5(20.83) | 0.330 |
| Hematuria, no (%) | 41（85.42） | | 24（100） | | 17（70.83） | 0.004 | 23(95.83) | 18(75) | 0.040 |
| Leukocyturia (noninfectious), no. (%) | 38（79.1） | | 22（91.67） | | 16（66.67） | 0.033 | 17（70.83） | 21(87.5) | 0.155 |
